# Course of COPD assessment test (CAT) and clinical COPD questionnaire (CCQ) scores during recovery from exacerbations of chronic obstructive pulmonary disease

**DOI:** 10.1186/1477-7525-11-147

**Published:** 2013-08-29

**Authors:** Marc Miravitlles, Patricia García-Sidro, Alonso Fernández-Nistal, María Jesús Buendía, María José Espinosa de los Monteros, Jesús Molina

**Affiliations:** 1 Pneumology Department, Hospital Universitari Vall d'Hebron, Ciber de Enfermedades Respiratorias (CIBERS), Barcelona, Spain; 2 Pneumology Unit, Hospital De La Plana, Villarreal, Spain; 3 Medical Department, Takeda Farmacéutica España S.A, Madrid, Spain; 4 Pneumology Department, Hospital Universitario Infanta Leonor, Madrid, Spain; 5 Pneumology Department, Hospital Universitario Virgen de la Salud, Toledo, Spain; 6 Centro de Salud “Francia”, Fuenlabrada, Madrid, Spain

**Keywords:** COPD, Health status, CAT, CCQ, Exacerbations

## Abstract

**Introduction:**

COPD exacerbations have a negative impact on lung function, decrease quality of life (QoL) and increase the risk of death. The objective of this study was to assess the course of health status after an outpatient or inpatient exacerbation in patients with COPD.

**Methods:**

This is an epidemiological, prospective, multicentre study that was conducted in 79 hospitals and primary care centres in Spain. Four hundred seventy-six COPD patients completed COPD assessment test (CAT) and Clinical COPD Questionnaire (CCQ) questionnaires during the 24 hours after presenting at hospital or primary care centres with symptoms of an exacerbation, and also at weeks 4–6. The scores from the CAT and CCQ were evaluated and compared at baseline and after recovery from the exacerbation.

**Results:**

A total of 164 outpatients (33.7%) and 322 inpatients (66.3%) were included in the study. The majority were men (88.2%), the mean age was 69.4 years (SD = 9.5) and the mean FEV1 (%) was 47.7% (17.4%). During the exacerbation, patients presented high scores in the CAT: [mean: 22.0 (SD = 7.0)] and the CCQ: [mean: 4.4 (SD = 1.2)]. After recovery there was a significant reduction in the scores of both questionnaires [CAT: mean: -9.9 (SD = 5.1) and CCQ: mean: -3.1 (SD = 1.1)]. Both questionnaires showed a strong correlation during and after the exacerbation and the best predictor of the magnitude of improvement in the scores was the severity of each score at onset.

**Conclusions:**

Due to their good correlation, CAT and CCQ can be useful tools to measure health status during an exacerbation and to evaluate recovery. However, new studies are necessary in order to identify which factors are influencing the course of the recovery of health status after a COPD exacerbation.

## Introduction

Chronic Obstructive Pulmonary Disease (COPD) is a major cause of death in industrialized countries. The mortality rate of this disease is increasing and it is likely to become the third leading cause of death worldwide in 2020 [1,2]. COPD is often aggravated by acute periods of increased symptoms called exacerbations. These are the most common reason for doctor’s visits, emergency department visits, hospital admissions and deaths [3].

In addition, numerous studies have shown that exacerbations generate a large impact on health systems [3,4]. For example, they cause 10-12% of primary care visits, 1-2% of emergency room visits and 10% of hospitalizations in Spain [4]. This is why, from an economic standpoint, these data are of particular relevance since 44% of the annual cost per patient involving COPD is due to hospital admissions, exacerbations being the most frequent cause [5].

Among many other aspects, exacerbations are associated to a deterioration in the patients’ quality of life [6]. However, studies that have focused on determining the evolution of a patient after suffering an exacerbation are mainly based on investigating functional [7,8] or inflammatory changes [9-11].

Certain generic quality-of-life questionnaires such as the EuroQol Five-Dimensional Questionnaire (EQ-5D) [12], or respiratory–specific questionnaires such as the St. George’s Respiratory Questionnaire (SGRQ) [13], may be useful to assess the recovery of patients who have suffered an exacerbation. However, the usefulness of the most widely used short questionnaires, the COPD Assessment Test (CAT) [14] and the Clinical COPD Questionnaire (CCQ) [15] to assess the course of moderate or severe exacerbations has not been investigated adequately. These two questionnaires have the great advantage of simplicity for the patient, both having a very good correlation with the SGRQ [14-16], considered the gold standard of specific health-related quality-of- life (HRQoL) questionnaires for COPD.

In this work we present the results of the course of CAT and CCQ scores during the recovery of an acute exacerbation of COPD and the factors associated to the changes in scores of patients recovering from these episodes, as well as the comparison and correlation between both instruments.

## Method

### Study design

The ECO study (Exacerbations and quality of life in COPD) was an observational, multicentre, prospective study aimed at evaluating the predictive value of different HRQoL questionnaires in the long-term course of patients measured as time to the next exacerbation or death. In order for the study to be feasible, we selected a population of patients after recovery from an exacerbation, because these subjects are more likely to suffer a second episode during follow-up [17]. Patients were recruited upon presentation at the hospital or at primary care offices with symptoms of an exacerbation. Those who met inclusion and exclusion criteria were informed about the study and were asked to sign an informed consent document. The physicians in charge collected information upon presentation or during the first 24 hours after admission. This information included demographic data, as well as data related to smoking, medical history and comorbidities. The cardiovascular risk was assessed according to BMI, gender and waist circumference [18]. Patients were asked to fill in COPD assessment test (CAT) and Clinical COPD Questionnaire (CCQ) quality-of-life questionnaires in their validated versions in Spanish.

The patients were evaluated again after 4 to 6 weeks from the initial visit, at which point they filled out both questionnaires once more. For the study, only those patients who recovered from the exacerbation during that time frame were followed up for time to next exacerbation or for death and they comprise the population of this analysis. The investigator had to verify clinical recovery; in addition, patients had to answer “somewhat better” or “much better” to a question about their health status: “In relation to the previous visit, how are you?” using a 5-point Likert scale with the following response options: “Much worse, worse, same, better, much better. ”Furthermore, the CAT score must have improved by at least 5 units to consider that the patient had recovered from the exacerbation [19].

Moderate COPD exacerbations were defined as a sudden increase in respiratory symptoms that required ambulatory treatment with systemic corticosteroids and/or antibiotics, and exacerbations were considered severe when the patient required hospitalization.

The study was approved by the ethics committee of the Hospital Clinic (Barcelona, Spain. Reference number 2012/5918) and all participants provided a written informed consent.

### Population

Patients of both genders, aged 40 years or older, were recruited in the study if they met the following inclusion criteria: a) COPD demonstrated by spirometry performed in stable state not more than 12 months before being recruited in the study with a post-bronchodilator ratio of FEV1/FVC < 0.7; b) smoker or former smoker of at least 10 pack-years; c) exacerbation defined as an increase in respiratory symptoms that requires treatment with systemic corticosteroids, antibiotics or both, and/or hospitalisation. On the other hand, the exclusion criteria in the study were: (i) patients with another chronic respiratory disease (e.g. bronchial asthma, cystic fibrosis, severe bronchiectasis, cancer, restrictive lung disease, etc.), (ii) patients with a COPD exacerbation due to other causes such as pneumonia, pneumothorax and decompensated congestive heart failure, (iii) patients requiring invasive or non-invasive mechanical ventilation (iv) patients who, in the opinion of the investigator, did not retain sufficient cognitive capacity, presenting sensory or psychiatric disability or language barriers that prevent or hinder a normal conduction of the study, (v) patients participating in another study or clinical trial.

### Measurements

The CAT consists of 8 items with scores ranging from 0 to 5 (0 = no impairment). An overall score is calculated by adding the score from each item with total scores ranging from 0 to 40, higher scores indicating a more severe health status impairment or a poorer control of COPD [16]. The CAT’s minimal clinical important difference (MCID) is not yet established and has been estimated at 3.76 points [20]. The CCQ has three domains: symptoms (4 items), functional status (4 items) and mental state (2 items), graded on a 7-point Likert scale from 0 to 6 (0 = no impairment) [14]. The MCID has been established at 0.41 points [21].

For the collection of information, a web-based form (e-CRF) has been designed specifically for this study.

### Statistical analysis

In order describe the qualitative variables, absolute frequencies and percentages were used; furthermore, for ordinal qualitative variables, cumulative frequencies and percentages were used. The description of quantitative variables was performed using the mean, standard deviation (SD), median and quartiles. A comparison of qualitative variables between two or more groups was performed using the chi-square test and/or Fisher’s exact test. A comparison of quantitative variables between two groups was performed using the Mann–Whitney U test or Student’s t test, depending on the distribution of the data. A comparison of quantitative variables between three or more groups was carried out using the Kruskall Wallis test or ANOVA, depending on the distribution of the data. The results of the analysis were adjusted by the techniques of Oldham and Blomqvist. The correlation between quantitative variables was performed using the Pearson correlation coefficient; a correlation was considered good when the R coefficient was higher than 0.7 and acceptable between 0.5 and 0.7.

We developed two regression models to determine the factors that could best explain the change in score in each of the questionnaires from the time of exacerbation until recovery. Due to the non-normal nature of the variable obtained for change in scores (recovery-exacerbation) for CAT, it was decided to analyse this variable as categorical by means of logistic regression analysis, divided into tertiles of change: improvement of 5–6 points; improvement of 7–12 points and improvement of more than 12 points. A linear regression analysis model for change in the CCQ was developed taking into account the variable as quantitative. Univariate analyses were carried out without considering an intercept in the model, since each intercept would vary depending on the type of each variable (e.g., there is no estimate of a mean population probability of intensity change in the CAT, but all the variability depends on the value of the dependent variable). The multivariate model, however, in addition to adjusting for the variables of the model, includes an intercept. For the analysis we used the SAS Enterprise Guide 4.3. (SAS v.9.2)

## Results

### Patient population

A total of 675 patients were recruited in the study. Of these, 45 were lost to follow-up, 39 did not meet inclusion or exclusion criteria, 28 did not recover from the exacerbation by 4 to 6 weeks and 77 were excluded due to insufficient information for analysis, leaving 486 (72%) eligible patients. The demographic characteristics of the patients are shown in Table 1. One hundred sixty-four (33.7%) were outpatients and 322 (66.3%) were inpatients. The majority were men (88.2%), the mean age was 69.4 years (SD = 9.5) and the mean FEV1 (%) was 47.7% (17.4%). Compared with outpatients, inpatients had a lower BMI, a longer smoking history and a lower cardiovascular risk. Regarding COPD, these patients had a longer history of the disease, as well as a greater number of exacerbations reported in the previous year and more severe lung function impairment as shown by spirometry.

**Table 1:** Demographic and clinical characteristics

**Variable**	**Total**	**Ambulatory**	**Hospital**	**p value**
	**N = 486**	**N = 164**	**N = 322**	
Sex, male	432 (88.9)	138 (84.2)	294 (91.3)	0.022
Age, years	69.4 (9.5)	69.5 (9.1)	69.3 (9.7)	0.91
BMI, Kg/m2	27.8 (9.7)	29.4 (14.9)	26.9 (5.1)	<0.001
Active smokers	139 (28.6)	49 (29.9)	90 (28)	0.65
Smoking habit, pack/years	54.5 (30.5)	48.9 (28.9)	57.3 (30.9)	0.004
Level of education				
*No school*	117 (24.1)	22 (13.4)	95 (29.5)	<0.001
*Primary education*	245 (50.4)	93 (56.7)	152 (47.2)	
*Secondary/higher education*	124 (25.5)	49 (29.9)	75 (23.3)	
Cardiovascular risk				
*None*	123 (25.3)	28 (17.0)	95 (29.5)	0.009
*Increased*	128 (26.3)	45 (27.4)	83 (25.8)	
*High*	115 (23.7)	39 (23.8)	76 (23.6)	
*Very high*	120 (24.7)	52 (31.7)	68 (21.1)	
Diabetes mellitus	127 (26.1)	41 (25.0)	86 (26.7)	0.68
Waist circumference, cm	99.2 (18.7)	101.3 (14.7)	98.1 (20.4)	<0.001
Time walking per day, minutes	55.0 (26.0)	68.2 (32.3)	48.08 (24.2)	<0.001
Time of COPD evolution, years	9.9 (8.3)	8.6 (7.4)	10.6 (8.6)	0.013
Number of exacerbations previous year	2.9 (2.7)	2.4 (1.9)	3.2 (2.9)	0.002
FVC, mL	2549.1 (795.9)	2930.9 (864.8)	2357.0 (683.0)	<0.001
FVC, %	68.1 (17.5)	76.3 (16.1)	64.0 (16.7)	<0.001
FEV1, mL	1312.9 (523.8)	1658.9 (534.9)	1138.8 (422.5)	<0.001
FEV1, %	47.7 (17.4)	59.3 (16.2)	41.8 (14.9)	<0.001
FEV1/FVC, %	51.4 (11.7)	56.9 (9.8)	48.7 (11.6)	<0.001

Values are expressed as mean (standard deviation) or frequency (%).

Footnote: *BMI* Body Mass Index.

### Changes in health status

**Figure 1:**
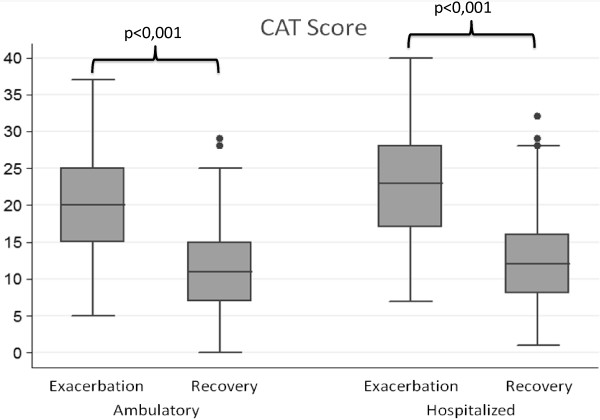
Course of CAT scores following an outpatient or inpatient exacerbation.

At the time of exacerbation, the patients had high scores on the CAT: [22.0 (SD = 7.0)], with differences between inpatients [22.8 (SD = 7.0)], and outpatients [20.4 (SD = 6.9)]. After recovery, there was a significant reduction of 9.9 (SD = 5.1) points. Improvements were significant in both inpatients and outpatients [8.9 (SD = 4.6) and 10.4 (SD = 5.3) respectively; p < 0.001 for both groups], (Table 2, Figure 1).

**Table 2:** Course of health status, CAT and CCQ test scores

**Questionnaire**	**Exacerbation**	**Recovery**	**Change (R-E)**	**P value**
**CAT total**				
Total	22.0 (7.0)	12.1 (5.9)	−9.9 (5.1)	<0.001
Outpatient	20.4 (6.9)	11.5 (5.8)	−8.9 (4.6)	<0.001
Inpatient	22.8 (7.0)	12.4 (6.0)	−10.4 (5.3)	<0.001
**CCQ total**				
Total	4.4 (1.2)	3.1 (1.1)	−1.3 (0.9)	<0.001
Outpatient	3.8 (1.2)	2.8 (1.0)	−1.0 (0.9)	<0.001
Inpatient	4.6 (1.1)	3.2 (1.1)	−1.4 (1.0)	<0.001
**CCQ; symptoms**				
Total	4.7 (1.3)	3.1 (1.1)	−1.6 (0.7)	<0.001
Outpatient	4.4 (1.3)	3.1 (1.1)	−1.3 (0.9)	<0.001
Inpatient	4.9 (1.2)	3.1 (1.1)	−1.8 (1.0)	<0.001
**CCQ: functional**				
Total	4.1 (1.4)	3.1 (1.3)	−1.0 (1.2)	<0.001
Outpatient	3.4 (1.3)	2.6 (1.1)	−0.8 (0.5)	<0.001
Inpatient	4.5 (1.3)	3.3 (1.3)	−1.2 (0.7)	<0.001
**CCQ: mental**				
Total	4.1 (1.5)	2.9 (1.4)	−1.2 (1.1)	<0.001
Outpatient	3.6 (1.5)	2.7 (1.3)	−0.9 (0.9)	<0.001
Inpatient	4.4 (1.5)	3.1 (1.4)	−1.3 (1.1)	<0.001

Values are expressed as mean points (standard deviation).

**Figure 2:**
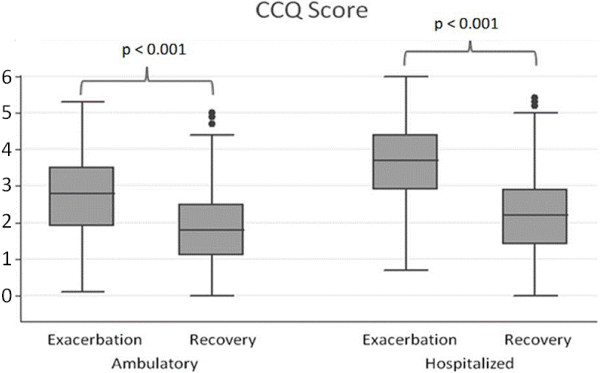
Course of CCQ scores following an outpatient or inpatient exacerbation.

Regarding the CCQ, the patients experienced a statistically significant improvement in the global scale after recovery, from a score of 4.4 points (SD = 1.2) at the time of exacerbation to 3.1 points (SD = 1.1) once stabilized. This improvement occurred similarly in outpatients and inpatients [1.0 (SD = 0.9) and 1.4 (SD = 1.0), respectively; p < 0.001 for both groups], and in all areas of the questionnaire, but particularly in the symptoms score (difference = −1.6 points (SD = 0.7); p < 0.001) (Table 2, Figure 2).

### Correlation between the questionnaires

**Figure 3:**
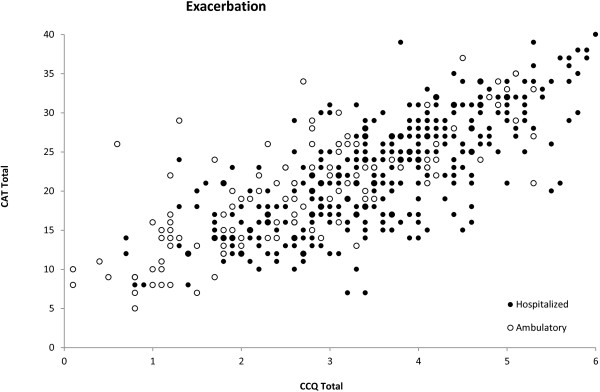
**Correlations between CAT and CCQ scores at onset of exacerbation.** Correlation of scores in the global population R = 0.748 (p < 0.0001), in outpatient exacerbations R = 0.788 (p < 0.0001) and in inpatient exacerbations R = 0.723 (p < 0.0001).

**Figure 4:**
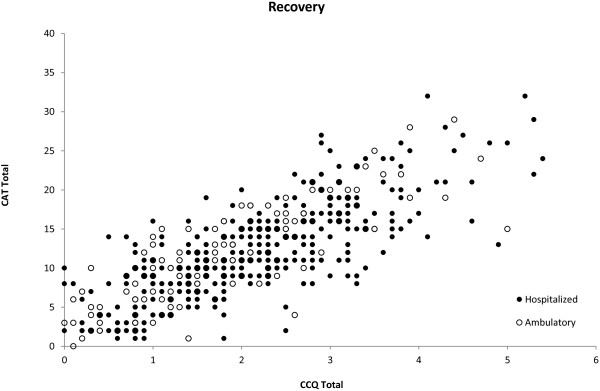
**Correlations between CAT and CCQ scores after recovery from the exacerbation.** Correlation of scores in the global population R = 0.780 (p < 0.0001), in outpatient exacerbations R = 0.827 (p < 0.0001) and in inpatient exacerbations R = 0.760 (p < 0.0001).

**Figure 5:**
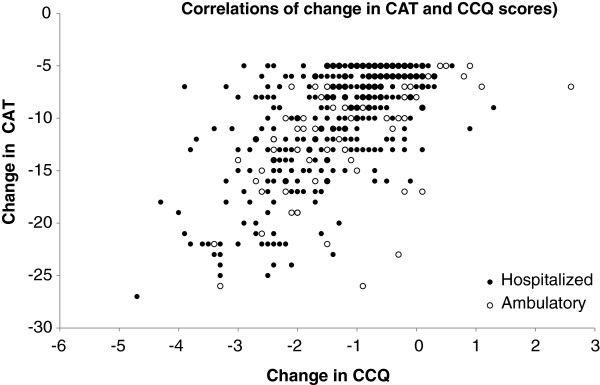
**Correlations between CAT and CCQ changes in scores between exacerbation and recovery.** Correlation of scores in the global population R = 0.594 (p < 0.001), in outpatient exacerbations R = 0.561 (p < 0.0001) and in inpatient exacerbations R = 0.591 (p < 0.0001).

Whatever the type of exacerbation (outpatient/inpatient), there was a good correlation between the CAT scores and the CCQ scores, both at exacerbation onset (R = 0.748; p < 0.0001) (Figure 3), and once the patient had recovered (R = 0.780; p < 0.001) (Figure 4). Furthermore, there was an acceptable correlation between the change (exacerbation-recovery) scores in both questionnaires (R = 0.594; p < 0.0001) (Figure 5).

### Predictors of changes in health status

After performing the logistic regression analysis, it was observed that the variation in the CAT scoring between exacerbation onset and recovery was greatly dependent on the score at exacerbation onset. Thus, there was a greater improvement for patients who began with the worse initial status [OR (95% CI): 0.84 (0.81 to 0.86), p < 0.001]. In this model, the outcome variable (change in CAT score) was categorised into 3 categories and the OR indicates the odds to move from each category to the next. Nevertheless, the adjusted model can only explain 32.4% of the variance (Table 3).

**Table 3:** Study of univariate and multivariate association of change in CAT score from exacerbation to stable state, categorized into three categories (5–6 points, 7–12 points, and over 12)

**Variable**	**Univariate**			**Multivariate**		
	**OR**	**95% CI**	**P**	**OR**	**95% CI**	**P**
Type of exacerbation (inpatient vs. outpatient RC)	2.3	1.6 to 3.2	<0.001			
Gender (men vs. women RC)	2.3	1.6 to 3.3	0.051			
Waist circumference	0.994	0.992 to 0.996	<0.001			
Smoking status (current vs. former smoker RC)	1.64	1.17 to 2.32	0.004			
Exacerbations previous year	0.89	0.85 to 0.93	<0.001			
FEV1 (%)	0.99	0.98 to 0.99	<0.001			
CAT at inclusion	0.96	0.95 to 0.97	<0.001	0.84	0.81 to 0.86	<0.001
CCQ at inclusion	0.84	0.80 to 0.87	<0.001			

Independent variables: waist circumference, exacerbations in the previous year, FEV1 (%) and CAT and CCQ at inclusion were continuous variables. The strength of the association is presented by the OR and 95% confidence interval obtained by logistic regression.

*OR* odds ratio, *CI* confidence interval, *RC* Reference category.

Regarding the CCQ, the resulting model explains 30.8% of the variability. The improvement in CCQ scores at recovery from exacerbation is greater when: there is a higher baseline CCQ score [β (95% CI): -0.46 (−0.52 to −0.40); p < 0.001], fewer total exacerbations in the past year [β (95% CI): 0.04 (0.01 to 0.07); p = 0.007] and the patient is not living with smokers [β (95% CI): -0.25 (−0.44 to −0.06); p = 0.01] (Table 4).

**Table 4:** Study of univariate and multivariate association of change in CCQ score from exacerbation to stable state

**Variable**	**Univariate**			**Multivariate**		
	**B**	**95% CI**	**P**	**B**	**95% CI**	**P**
Type of exacerbation (inpatient vs. outpatient RC)	−1.44	−1.56 to −1.32	<0.001			
Gender (men vs. women RC)	−1.27	−1.38 to −1.17	<0.001			
BMI	−0.004	−0.008 to −0.0002	<0.001			
Smoking (current vs. former-smoker RC)	1.33	1.57 to 1.09	<0.001			
Lives with smokers (yes vs. no RC)	1.44	1.12 to 1.75	<0.001	−0.25	−0.44 to −0.06	0.010
Exacerbations previous year	−0.25	−0.28 to −0.22	<0.001	0.04	0.01 to 0.07	0.007
FEV1 (%)	−0.023	−0.043 to 0.003	<0.001			
CAT at inclusion	−0.057	−0.06 to −0.05	<0.001			
CCQ at inclusion	0.84	0.80 to 0.87	<0.001	−0.46	−0.52 to −0.04	<0.001

The CCQ score change has been studied as a continuous variable and the strength of the association occurs through the beta coefficients obtained by linear regression. BMI, exacerbations previous year FEV1 (%) and CAT and CCQ at inclusion were continuous variables.

*B* Beta coefficient, *CI* confidence interval, *RC* Reference category.

## Discussion

The results of the current study have demonstrated there is a severe impairment in the health status of exacerbated patients, both in inpatient and outpatient settings. As expected, scores of both questionnaires were significantly worse in admitted compared with ambulatory patients. The patients who recovered from the acute episode showed a highly significant improvement in the scores of the CAT and the CCQ. Furthermore, there was a good correlation of the scores of both questionnaires at exacerbation onset and at recovery. The best predictor of the magnitude of the improvement in scores of both questionnaires is the score of the given questionnaire at exacerbation onset. These results suggest that both questionnaires can be used in a clinical setting to evaluate the improvement in health status during and/or after the treatment of a moderate or severe exacerbation of COPD.

The CAT questionnaire has shown to provide significantly different scores in stable and exacerbated patients in cross-sectional studies. Jones et al. [22], in a validation study, reported a mean score of 17.2 points in stable COPD patients compared with 21.3 in exacerbated COPD patients. Agustí et al. [23] obtained a score of 22.4 points in a group of COPD inpatients compared with 15.8 in stable patients. Interestingly, we obtained an almost identical CAT score in our inpatients (22.8 points), which was significantly reduced to only 12.4 after recovery from the acute episode. This large improvement in CAT scores after an admission is similar to the one reported by the previous group of investigators in patients who claimed to feel “much better” after recovery from the hospitalization (8.9 units) [13]. When analysing the data in the opposite direction (from stable state to exacerbation) MacKay et al. [19] noted a change from 19.4 to 24.1 points in the CAT score in a population of outpatients with COPD. This change was smaller in magnitude to the one observed in our study, but the health status impairment of those patients was more severe than that observed in our population (CAT = 12.1 at recovery). Another difference is that return to baseline in the aforementioned study was considered at 11 days, in contrast to 4 to 6 weeks in our study. Previous studies using the SGRQ suggested that the health status may require up to 12 weeks to full recovery [13]. Despite of these results, we have to take into consideration the fact that CAT has not been validated for determining the severity of an exacerbation. In fact, some subjects included in the Mackay [19] study had a decline in the CAT score when they suffered an exacerbation, while others had no significant changes. Questionnaires designed specifically to assess the changes in health status during exacerbations such as the EXAcerbations of Chronic Obstructive Pulmonary Disease Tool (EXACT) can be more useful for quantifying the severity of these events [24].

In addition, it must be noted that due to the design of the study, we only considered those patients who recovered from the exacerbation and one of the criteria for recovery was an improvement of at least 5 points in CAT scores. Therefore, we cannot analyse the validity of the questionnaires to identify patients who could fail in the treatment of an exacerbation. However, only 28 patients (4.2%) could not be recruited in the study because they did not reach this threshold at recovery.

Regarding the CCQ, we noted a mean reduction of −1.3 points at recovery, which was even higher for the symptoms scale (−1.6 points) and clearly higher than the suggested clinically significant minimum difference of 0.41 [20]. In a previous cross-sectional study on 3,935 COPD patients in Spain, the mean value of CCQ was 2.5 and it was significantly associated to the degree of dyspnoea, the frequency of previous exacerbations and hospitalizations, the severity of FEV1 impairment and a low level of education and physical activity [25]. However, longitudinal data of CCQ during recovery from exacerbations is limited. In contrast, an improvement in total scores of around 0.50 points has been observed after quitting smoking (improvement of 1.02 points in symptoms) [14,26]. The CCQ scores have been used for monitoring patients to predict treatment failure or the occurrence of an exacerbation. Using weekly measurements, an impairment of 0.20 points had a positive predictive value of 43.5% and a negative predictive value of 90.8% for the onset of an exacerbation in the next week [27]. Furthermore, a recent work in which the patients were monitorised daily during an exacerbation, showed that the absence of improvement in CCQ symptoms score and impaired lung function were independent predictors of treatment failure [28]. Concurrent with our results, the authors find the rate and pattern of recovery very similar in outpatients and inpatients [28].

Similarly to CAT, the best predictor of a large improvement in CCQ score after recovery was a worse score at exacerbation onset. A regression to the mean effect cannot be ruled out; however, for the improvement in CCQ scores other variables showed a significant and independent association with the magnitude of change. The patients who experienced fewer exacerbations in the past had a higher improvement in CCQ scores, as did those who lived with smokers. The relationship between repeated exacerbations and impairment in HRQoL has been noted in numerous studies [6,29], and was seen consistently with our results; a previous study demonstrated that patients who suffered repeated exacerbations did not revert to the baseline values of health status after an exacerbation [13]. The relationship between a higher improvement in CCQ scores and living with smokers is difficult to account for and might be related with worse scores at onset because more severe symptoms associated to second hand smoke, but this hypothesis should be tested adequately. However, only a small part of the variance has been explained by the variables included in the regression models. Other factors not addressed in our study may influence the recovery of these patients. For instance, Papaioannou et al. [30] observed that depressive symptoms had a negative effect on CAT improvement during recovery from an exacerbation.

The difference between the predictors of recovery found for CAT and CCQ can be explained in part by the different types of analysis; however, there are also some differences between them. CAT is a one-dimensional questionnaire, in turn, CCQ has different domains that correlate better with the corresponding SGRQ domains [20]. Furthermore, CCQ has been validated to be used at the level of individual patients [31].

More interesting and unique in our results was the relationship between the scores of both questionnaires. We have demonstrated a good correlation of the scores of CAT and CCQ at exacerbation onset and at recovery and also an acceptable correlation of the changes in scores observed in both questionnaires after recovery. These results suggest that both are measuring the same effects and that both can be used reliably in this context. To the best of our knowledge, this is the first study to compare the performance of both questionnaires during the recovery of outpatient and inpatient exacerbations. The CAT and the CCQ have also demonstrated a good correlation in stable COPD; Tsiglianni et al [20] observed a rho = 0.64 between both scores in a group of 90 stable COPD patients over three repeated measurements. Moreover, in a study of severe COPD patients referred for rehabilitation, a correlation of r = 0.77 was shown between the CAT and the CCQ [32]. These values are similar to those obtained in our patient population ranging from r = 0.72 to r = 0.82 for correlations between both questionnaires either at onset or at recovery from the exacerbation. These results support the use of either questionnaire in the clinical assessment of patients with COPD, as suggested in recent guidelines [33,34].

Our study has several limitations, we have only analysed the patients that recovered from an exacerbation and therefore we cannot investigate a different course of scores according to clinical outcomes. Our results describe only the course of improvement of health status after an exacerbation. Another limitation is the use of a CAT cut-off value of 5 points for defining recovery from an exacerbation. Currently, there is no well-established MCID for CAT, which is why we also used the investigator’s clinical judgement and a question with a Likert scale for the patient to contribute to the definition of recovery.

One of the strengths of our study is its observational design in a large sample of patients in different settings, which allows for the extrapolation of results with a high external validity.

In summary, our results have demonstrated a severe impairment in the health status of COPD patients at exacerbation onsets, as measured by CCQ and CAT scores. Health status significantly improves during recovery. There is a strong correlation between the scores of both questionnaires at onset and at recovery and the best predictor of the magnitude of improvement is the severity of the score at onset. Both instruments can be used to monitor the course of recovery of outpatient or inpatient COPD exacerbations.

## Competing interests

Marc Miravitlles has received speaker fees from Boehringer Ingelheim, Pfizer, AstraZeneca, Bayer Schering, Novartis, Talecris-Grifols, Takeda-Nycomed, Merck, Sharp & Dohme and Novartis, and consulting fees from Boehringer Ingelheim, Pfizer, GSK, AstraZeneca, Bayer Schering, Novartis, Almirall, Merck, Sharp &Dohme, Talecris-Grifols and Takeda-Nycomed. Alonso Fernández is a full time employee of Takeda. The rest of authors have no conflicts of interest to disclose.

## Authors’ contributions

MM, JM and AFN designed the study and directed the data analysis. MM and AFN drafted the manuscript. PGS, MJB, MJEM, JM participated in data collection and critical revisión of the data analysis. All authors read and approved the final manuscript.
